# Plasma Protein Profiling Reveals Protein Clusters Related to BMI and Insulin Levels in Middle-Aged Overweight Subjects

**DOI:** 10.1371/journal.pone.0014422

**Published:** 2010-12-23

**Authors:** Susan J. van Dijk, Edith J. M. Feskens, A. Geert Heidema, Marieke B. Bos, Ondine van de Rest, Johanna M. Geleijnse, Lisette C. P. G. M. de Groot, Michael Müller, Lydia A. Afman

**Affiliations:** 1 Division of Human Nutrition, Wageningen University, Wageningen, The Netherlands; 2 The Netherlands Nutrigenomics Centre, TI Food and Nutrition, Wageningen, The Netherlands; Institute of Preventive Medicine, Denmark

## Abstract

**Background:**

Biomarkers that allow detection of the onset of disease are of high interest since early detection would allow intervening with lifestyle and nutritional changes before the disease is manifested and pharmacological therapy is required. Our study aimed to improve the phenotypic characterization of overweight but apparently healthy subjects and to identify new candidate profiles for early biomarkers of obesity-related diseases such as cardiovascular disease and type 2 diabetes.

**Methodology/Principal Findings:**

In a population of 56 healthy, middle-aged overweight subjects Body Mass Index (BMI), fasting concentration of 124 plasma proteins and insulin were determined. The plasma proteins are implicated in chronic diseases, inflammation, endothelial function and metabolic signaling. Random Forest was applied to select proteins associated with BMI and plasma insulin. Subsequently, the selected proteins were analyzed by clustering methods to identify protein clusters associated with BMI and plasma insulin. Similar analyses were performed for a second population of 20 healthy, overweight older subjects to verify associations found in population I. In both populations similar clusters of proteins associated with BMI or insulin were identified. Leptin and a number of pro-inflammatory proteins, previously identified as possible biomarkers for obesity-related disease, e.g. Complement 3, C Reactive Protein, Serum Amyloid P, Vascular Endothelial Growth Factor clustered together and were positively associated with BMI and insulin. IL-3 and IL-13 clustered together with Apolipoprotein A1 and were inversely associated with BMI and might be potential new biomarkers.

**Conclusion/ Significance:**

We identified clusters of plasma proteins associated with BMI and insulin in healthy populations. These clusters included previously reported biomarkers for obesity-related disease and potential new biomarkers such as IL-3 and IL-13. These plasma protein clusters could have potential applications for improved phenotypic characterization of volunteers in nutritional intervention studies or as biomarkers in the early detection of obesity-linked disease development and progression.

## Introduction

Cardiovascular disease (CVD) and type 2 diabetes (T2DM) are common disorders affecting millions of people worldwide. Evidence is accumulating that chronic low-grade inflammation plays a role in the development of both diseases [1], [2]. Increased plasma levels of several pro-inflammatory proteins and decreased levels of anti-inflammatory proteins have been observed in subjects with obesity and obesity-related diseases such as CVD and T2DM [3], [4], [5], [6].

Certain pro-inflammatory plasma proteins are used as diagnostic biomarkers for disease state but specific plasma proteins may also be used as biomarkers for early state in the development of a disease. Such an improved pre-disease diagnostic would allow intervening with relatively mild strategies such as lifestyle interventions with specific dietary regimes and increased physical activity in contrast to pharmacological therapy required once the disease is manifested. Identification of biomarkers that allow detection of the onset of disease will help in prevention of the disease. Plasma proteins might be good candidates as they circulate throughout the whole body, thereby reflecting total body metabolic and inflammatory status. Moreover, blood can be easily obtained from human subjects and therefore plasma proteins can be easily measured for screening purposes.

So far, in most studies that investigated the use of plasma proteins as biomarkers only a few plasma proteins were measured. However, the etiology of diseases such as CVD and T2DM is complex and the measurement of multiple biomarkers will provide additional information about the individual phenotype and health status as compared with measurement of a single biomarker [7], [8]. Recent technological advances such as multiplex immunoassays allow for the measurement of over hundred proteins at a time in one small plasma sample. Identification of biomarker profiles in such large protein datasets requires advanced statistical analyses. Random Forest (RF) has shown to be suitable for analysis of complex data sets as derived from proteomics analysis [9], [10]. RF is a technique that can prioritize and select from a large number of variables a set of variables that is likely to be related to the outcome of interest. Furthermore, in the prioritization and selection process, it provides a way to take interactions between proteins into account [11], [12]. The proteins that are selected by RF can subsequently be analyzed by clustering methods, offering the opportunity to identify clusters of proteins that are associated with different health outcomes.

Our study aimed to improve the phenotypic characterization of overweight but apparently healthy subjects and to identify new candidate profiles for early biomarkers of obesity-related diseases such as CVD and T2DM.

## Methods

### Subjects

Two populations were included in this study; population I was the primary study population of interest and population II was a smaller population used for verification of the results found in population I.

Population I consisted of 56 healthy men and women who participated in a controlled feeding trial [13]. Subjects included were aged 40–65 years with a BMI≥25 kg/m^2^ or a waist circumference ≥94 cm for men and ≥80 cm for women. Excluded were hypercholesterolemic subjects (fasting total cholesterol ≥8 mmol/L) and subjects with non-treated diabetes mellitus (according to WHO criteria) as measured by an oral glucose tolerance test during screening. Other exclusion criteria were the use of serum lipid or blood pressure lowering medication.

Population II consisted of 20 healthy, independently living elderly men and women. This population is a subgroup of the population participating in the study of Van de Rest et al. [14]. Subjects included were aged >65 years without depression, dementia or serious liver disease. This population was chosen because subjects were healthy and had average BMI and insulin values comparable to those from population I.

Both studies were approved by the Medical Ethics Committee of Wageningen University and all subjects gave written informed consent.

### Plasma proteins

In population I, concentrations of 124 proteins, including insulin, were measured in fasting plasma by quantitative multiplex immunoassay based on Luminex xMAP technology (Rules Based Medicine Inc, Austin, Texas, USA) according to the procedure described by Domenici et al. [15]–[16]. For each multiplex, both calibrators and controls were included on each microtiter plate. 8-point calibrators were run in the first and last column of each plate and 3-level controls were included in duplicate. Testing results were determined first for the high, medium and low controls for each multiplex to ensure proper assay performance. Unknown values for each of the analytes localized in a specific multiplex were determined using 4 and 5 parameter, weighted and non-weighted curve fitting algorithms included in the data analysis package. The plasma samples were run in duplicate and data reported as concentrations (average of two independent measures), together with data for the least detectable dose (LDD). Any value above the LDD will possess coefficients of variation (CV) less than 20%. Rules-Based Medicine's Multi-Analyte Profiles have been validated to Clinical Laboratory Standards Institute guidelines.

The set of proteins present on the assay consists of factors that are implicated in chronic diseases, inflammation, endothelial function and metabolism. In table S1 all proteins measured are listed.

Before analysis 16 out of 124 proteins (figure S1) were removed from the dataset as the concentrations of these proteins were below the detection limit in more than half of the samples. For the remaining 108 proteins, values below the detection limit were replaced by 0.1*Least Detectable Dose. As IL-6 was one of the removed proteins and IL-6 is an important factor for obesity and diabetes [6], this protein was separately measured by high-sensitive enzyme immunoassay (Human IL-6 Quantikine HS ELISA Kit, R&D Systems, Abingdon, United Kingdom). Apo lipoprotein B (ApoB) was not included in the Human Multi-Analyte Profiles, and ApoB levels were additionally measured on a Hitachi 912 autoanalyser (Roche, Lelystad, The Netherlands) using a commercially available kit (Roche cat. nr.1551779). In population II, the plasma concentrations of 107 out of the 124 proteins measured in population I, were determined using multiplex immunoassay (Rules Based Medicine Inc, Austin, Texas, USA) (table S1). In total, 110 proteins were included in the analysis for population I and 90 proteins were included in the analysis for population II (figure S1).

### Statistical analyses

#### Univariate analyses

The association between individual protein concentrations, BMI and plasma insulin concentration was calculated by univariate analysis. The statistical package PASW (version 17.0; SPSS, Chicago, IL) was used for the univariate analysis. Since the distribution of several variables was slightly skewed in the population, Spearman correlation coefficients were calculated for the association between protein concentrations, BMI and insulin concentrations.

#### Random Forest

Random Forest (RF) was used to provide a ranking in the importance of proteins in their relationship with BMI as well as with plasma insulin concentrations, taking possible interactions between proteins into account. The R package randomForest (R-package randomForest, http://cran.r-project.org/), which is based on the original FORTRAN code from Breiman et al. [11] was used for the analysis (www.stat.berkeley.edu/~breiman/RandomForests/).

In RF a group of tree-based models (the forest) is used to rank the proteins with an important contribution to BMI or insulin values [12], [17]. Each tree starts with the total data set, which is split into smaller and more homogeneous groups to fit models for predicting the outcome from the measured proteins. Within the forest, different trees are obtained by bootstrap sampling and random subset selection.

Importance of proteins in association with the outcome of interest is defined by a measure referred to as the importance index, I_m_. For each protein, this I_m_ is obtained by comparing the predictive performance of the forest for all proteins with the predictive performance of the forest in which the values of the protein are randomly permuted in the trees for the left-out observations. Larger differences in the predictive performance give a larger I_m_, indicating that the protein is more important. By permuting the values for one protein, not only the effect of this protein is taken into account, but also all possible interactions of this protein with other proteins. Interactions between proteins increase the I_m_ for each of the proteins that are part of the interaction. Thus, in the ranking of proteins by their importance RF takes interactions between proteins into account.

To perform the RF analyses we used the scaled mean decrease in prediction accuracy. To obtain stable estimates of the I_m_ and to capture as many important interactions as possible, the analyses were performed with a large number of trees (40,000). I_m_ was used as measure to rank the proteins. We chose not to apply a FDR estimation of the Im scores, because FDR estimation of importance scores derived by tree-based approaches usually overestimates the real FDR it can lead to an unreliable selection of a subset of variables [18].

For RF analysis a threshold value of significance does not exist. In this study a threshold was set at an I_m_ of 5 and only proteins with an I_m_>5 were considered for subsequent cluster analyses. We chose for this liberal threshold to avoid the possibility of leaving out proteins that might be of importance in relation to BMI and insulin.

### Clustering of the proteins

The program MultiExperimentViewer, version 4.3 was used for hierarchical clustering and visualization of the data [19]. Hierarchical clustering organizes the data into a binary tree that groups similar elements together. Proteins, BMI and insulin were clustered based on their Spearman correlation coefficients to select groups of proteins with high correlation with BMI or insulin and with each other. Besides clustering of the proteins with BMI and proteins with insulin based on their correlation coefficients also individual protein profiles were clustered based on similarities in protein concentrations. To compare the individual protein concentrations, z-scores were calculated for each individual protein 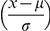.

To compare the association of the identified clusters of proteins and BMI to the association of single traditional biomarkers and BMI regression analysis was performed using the statistical package PASW (version 17.0; SPSS, Chicago, IL).

### Pathway analysis

Ingenuity Pathways Analysis, version 8.7 (Ingenuity® Systems, www.ingenuity.com) was used to identify connections between proteins and canonical pathways and diseases that were most significant to the data. Proteins were entered in Ingenuity based on their Swiss Prot ID and only connections, both direct and indirect, between proteins for humans and human primary cells were considered in the analysis.

## Results

### Baseline characteristics

Characteristics of the two study populations used in the analysis are displayed in table 1. BMI, waist circumference, insulin levels and percentage smokers were comparable in the two populations and age was significantly higher (13.7±1.1) in population II.

**Table 1:** Characteristics of the study populations.

	Population I (n = 56)	Population II (n = 20)
Sex (m/f)	23/33	10/10
Age (years) *	56.4±6.7	70.1±3.1
BMI (kg/m^2^)	27.4±5.0	28.3±5.4
Smokers (%)	11%	10%
Waist circumference (cm)	96.9±13.2	99.0±13.8
Insulin (pmol/L)	16.11±8.84	14.24±8.05

Data is displayed as mean ± standard deviation.

*Significantly different between the populations, p<0.05.

Plasma protein concentrations for both populations are displayed in table S2.

### Associations of proteins with BMI

**Figure 1:**
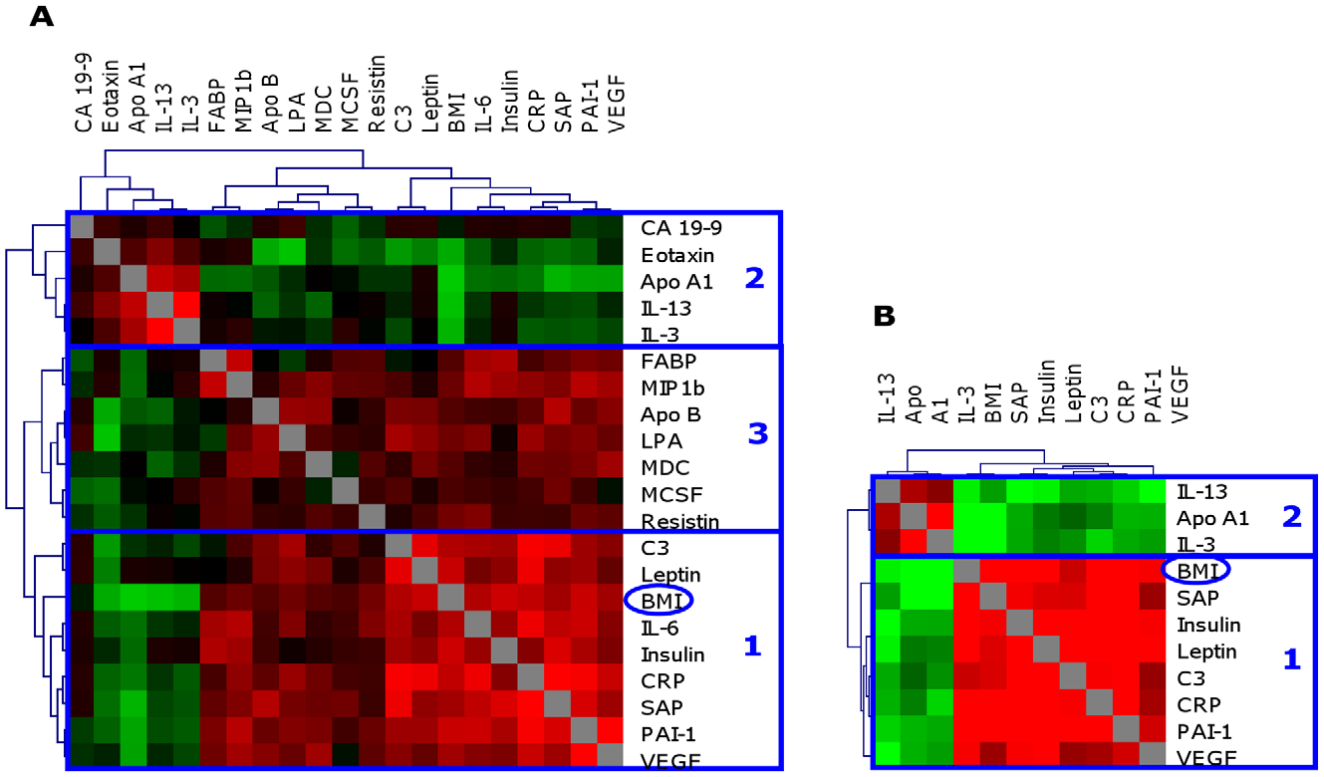
Correlation matrix of selected proteins associated with BMI in population I (A) and II (B). Correlation coefficients are displayed on a color scale, ranging from −0.6 (green) to 0.6 (red). For population I (A) 20 proteins selected by RF to be associated with BMI are included. This group of proteins was divided in three clusters based on similarity in the association with BMI and with each other. For population II (B) 10 proteins selected by RF and also associated with BMI in population I are included. This group of proteins was divided in two clusters based on similarity in the association with BMI and with each other. Abbreviations: Cancer Antigen 19-9 (CA 19-9), Apolipoprotein A1 (ApoA1), Fatty Acid Binding Protein (FABP), Macrophage Inflammatory Protein-1 beta (MIP1b), Apolipoprotein B (ApoB), Lipoprotein A (LPA), Macrophage Derived Chemokine (MDC), Macrophage colony-stimulating factor (MCSF), Complement 3 (C3), C Reactive Protein (CRP), Serum Amyloid P (SAP), Plasminogen Activator Inhibitor-1 (PAI-1), Vascular endothelial growth factor (VEGF).

In population I the RF I_m_ of 20 proteins was above 5 and these proteins were considered to be associated with BMI (table 2). Using univariate analysis, 14 out of these 20 selected proteins correlated significantly with BMI (table 2). For these 20 proteins and BMI mutual correlation coefficients were calculated. Based on these correlation coefficients a correlation matrix was constructed in which the proteins and BMI were clustered by similarity in their correlations (figure 1A). Using this approach, three clusters of proteins associated with BMI could be identified; cluster 1 and 3 were positively associated with BMI while cluster 2 was negatively associated with BMI. Cluster 1 showed robust associations with BMI and contains proteins highly positively associated with BMI and with each other and included insulin, leptin, Complement 3 (C3), Interleukin 6 (IL-6), C Reactive Protein (CRP), Plasminogen Activator Inhibitor (PAI-1), Serum Amyloid P (SAP) and Vascular Endothelial Growth Factor (VEGF). Cluster 2 also showed robust associations with BMI and the included proteins were inversely associated with BMI and positively with each other (figure 1A) and contained the proteins Apolipoprotein A1 (ApoA1), Cancer Antigen 19-9 (CA 19-9), Eotaxin, IL-3 and IL-13. The third cluster includes proteins that were positively associated with BMI and each other but most of these associations were less pronounced than in clusters 1 and 2.

**Table 2:** Association of proteins with BMI in the two populations.

	Population I (n = 56)		Population II (n = 20)	
Protein	I_m_ RF	Spearman correlation	I_m_ RF	Spearman correlation
1. Leptin	71.8	0.47 **	39.4	0.76 **
2. MCSF	49.1	0.18	n.d.	n.d.
3. IL-3	42.8	−0.43 **	11.7	−0.67 **
4. Insulin	35.4	0.48 **	51.4	0.74 **
5. Apo A1	33.0	−0.48 **	24.4	−0.66 **
6. IL-13	25.7	−0.46 **	26.1	−0.57 **
7. PAI-1	19.7	0.48 **	22.7	0.63 **
8. Eotaxin	17.2	−0.40 **	<5	−0.29
9. CA19-9	16.2	−0.06	<5	0.30
10. CRP	15.0	0.46 **	10.4	0.60 **
11. MDC	14.4	0.20	<5	0.01
12. Resistin	14.1	0.27 *	<5	0.37
13. IL-6	11.4	0.42 **	n.d.	n.d.
14. Apo B	11.1	0.20	n.d.	n.d.
15. SAP	8.6	0.38 **	34.6	0.70 **
16. C3	8.5	0.42 **	36.5	0.48 *
17. MIP1b	7.5	0.25	<5	0.31
18. VEGF	6.1	0.38 **	22.8	0.59 **
19. FABP	5.4	0.20	<5	0.11
20. LPA	5.2	0.26 *	<5	−0.11

*p<0.05, ** p<0.005.

Abbreviations: not determined (n.d.), Importance Index Random Forest (I_m_ RF), Macrophage colony-stimulating factor (MCSF), Apolipoprotein A1 (ApoA1), Plasminogen Activator Inhibitor-1 (PAI-1), Cancer Antigen 19-9 (CA 19-9), C Reactive Protein (CRP), Macrophage Derived Chemokine (MDC), Apolipoprotein B (ApoB), Serum Amyloid P (SAP), Complement 3 (C3), Macrophage Inflammatory Protein-1 beta (MIP1b), Vascular endothelial growth factor (VEGF), Fatty Acid Binding Protein (FABP), Lipoprotein A (LPA).

In population II 22 proteins were associated with BMI, based on RF I_m_ above 5, of which ten proteins were also associated with BMI in population I (table 2 and figure S1). A correlation matrix of these ten proteins and BMI was made for population II. Clustering of the proteins in population II was similar to the clustering in population I (figure 1A and 1B). The protein clusters 1 and 2, which showed robust associations in population I, were also associated with BMI in population II. The weaker associations of protein cluster 3 could not be confirmed. Associations in population II were more robust compared to associations in population I.

Regression analysis to compare the association of the identified clusters of proteins and BMI to the association of single traditional biomarkers such as CRP and IL-6 showed that the explained variance was higher when all proteins of cluster 1 were included in the model compared to when only IL6 or CRP or a combination of both were included. For BMI, the proportion of variance explained was 16.3% for IL-6 alone, 19.4% for CRP alone, 22.0% for CRP and IL-6 combined, and 32.3% when all proteins from cluster 1 were included in the model. For insulin, cluster 1 explained 25.6% of the variance, compared to 8.3% by CRP alone, 11.8% by IL-6 alone, and 12.0% by CRP and IL-6 combined.

**Figure 2:**
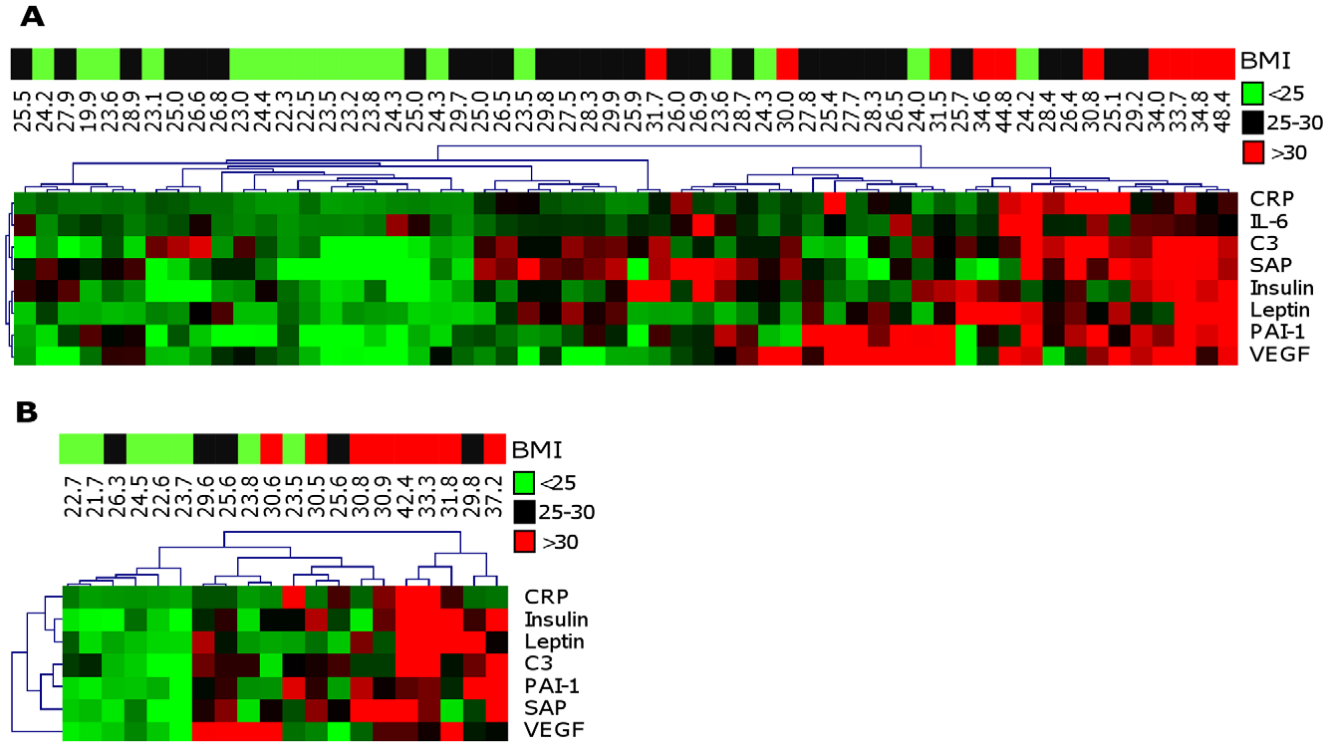
Clustering of personal profiles of robustly positively BMI-associated proteins in population I (A) and II (B). Protein values are displayed on a color scale; red indicates higher values than group average, green indicated lower values than group average. The personal BMI values are displayed on a separate color scale; green for BMI<25, black for BMI 25–30 and red for BMI>30. Only robustly positively BMI-associated proteins from cluster 1, as identified by RF and cluster analysis, were included in this figure.

Out of the cluster analysis we selected the highly BMI-associated proteins from cluster 1 to plot individual plasma profiles (figure 2). Subjects with similar plasma protein concentrations were clustered and their BMI values were subsequently displayed. Figure 2 shows that, as expected, in general persons with higher BMI have higher concentrations of the selected proteins. However, a few persons with BMI values <25 kg/m^2^ had high plasma levels of these proteins and a few persons with BMI values >30 kg/m^2^ had low plasma protein levels.

### Association of proteins with insulin concentration

**Figure 3:**
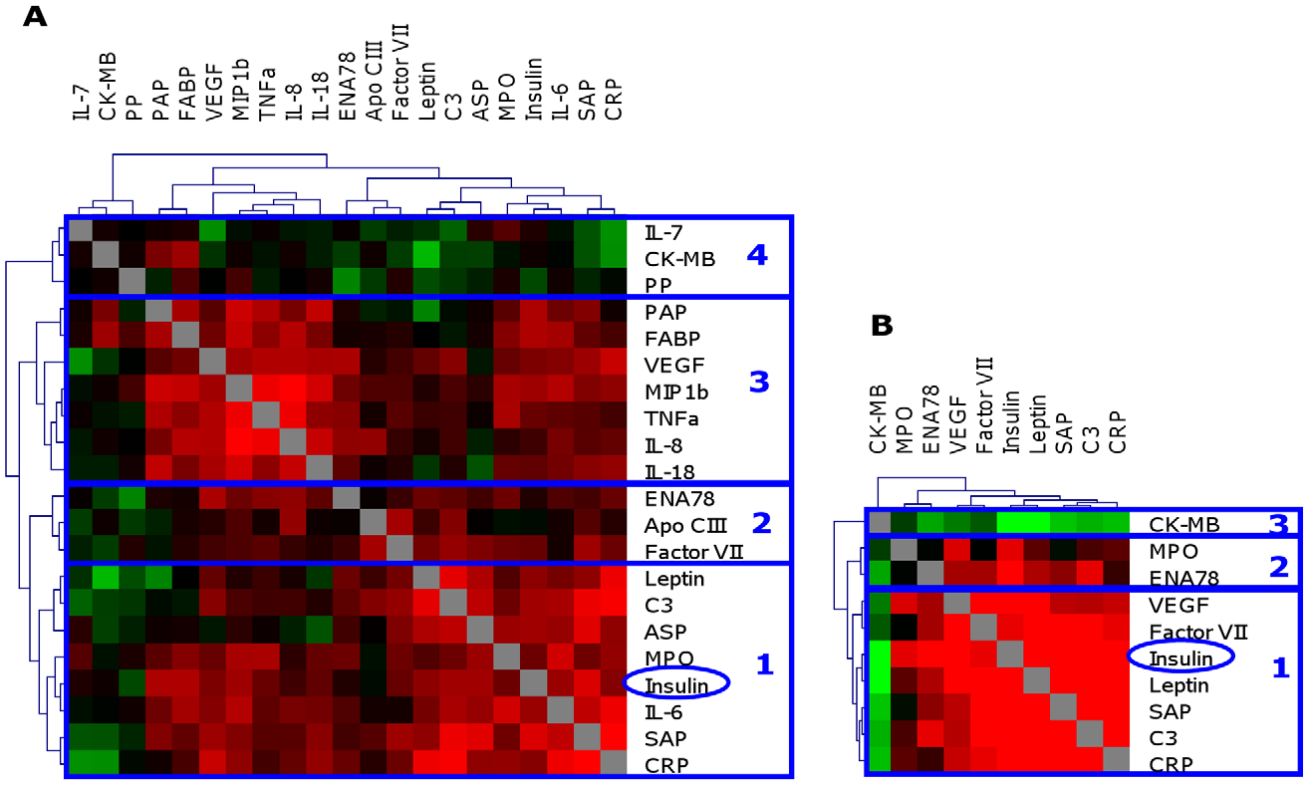
Correlation matrix of selected proteins associated with insulin in population I (A) and II (B). Correlation coefficients are displayed on a color scale, ranging from −0.6 (green) to 0.6 (red). For population I (A) 20 proteins selected by RF to be associated with fasting plasma insulin are included. This group of proteins was divided in four clusters based on similarity in the association with insulin and with each other. For population II (B) 9 proteins selected by RF and also associated with BMI in population I are included. This group of proteins was divided in three clusters based on similarity in the association with insulin and with each other. Abbreviations: Creatine Kinase-MB (CK-MB), Pancreatic Polypeptide (PP), Prostatic Acid Phosphatase (PAP), Fatty Acid Binding Protein (FABP), Vascular endothelial growth factor (VEGF), Macrophage Inflammatory Protein-1 beta (MIP1b), Tumor Necrosis Factor alpha (TNFα), Epithelial neutrophil-activating peptide 78 (ENA-78), Apolipoprotein CIII (Apo CIII), Complement 3 (C3), Acylation Stimulating Protein (ASP), Myeloperoxidase (MPO), Serum Amyloid P (SAP), C Reactive Protein (CRP).

The association between protein profiles and fasting insulin concentration was investigated using the same approach as for BMI. In population I, RF analysis identified 20 proteins that were considered to be associated with insulin concentration (table 3). Using univariate analysis, ten of these proteins significantly correlated with insulin concentration. Hierarchical clustering of the 20 selected proteins and insulin based on correlation coefficients resulted in the formation of four separate protein clusters (figure 3A). The proteins forming cluster 1, 2 and 3 were all positively associated with insulin concentration and the proteins in the fourth cluster were negatively associated with insulin.

**Table 3:** Association of proteins with fasting plasma insulin in both populations.

	Population I (N = 56)		Population II (N = 20)	
Protein	Im RF	Spearman correlation	Im RF	Spearman correlation
1. SAP	42.9	0.518**	25.8	0.579*
2. Leptin	32.2	0.333*	23.3	0.695**
3. PAP	29.2	0.411**	<5	−0.165
4. MIP1b	28.8	0.376**	<5	0.104
5. C3	18.5	0.379**	29.0	0.621**
6. IL-7	16.9	0.077	<5	−0.492*
7. FABP	16.8	0.416**	<5	0.099
8. ASP	16.0	0.383**	<5	0.220
9. VEGF	11.1	0.296*	21.3	0.590*
10. CK_MB	10.0	0.040	41.8	−0.614*
11. TNFα	9.9	0.250	<5	0.340
12. MPO	9.0	0.257	30.4	0.450*
13. IL-6	8.0	0.363**	n.d.	n.d.
14. PP	7.8	−0.171	<5	−0.219
15. Apo CIII	7.5	−0.240	<5	−0.072
16. ENA-78	7.2	0.082	5.5	0.520*
17. Factor VII	6.8	0.243	10.9	0.460*
18. IL-8	6.1	0.149	<5	0.155
19. IL-18	5.8	0.230	<5	−0.169
20. CRP	5.1	0.317*	36.6	0.723**

*p<0.05, ** p<0.005.

Abbreviations: Importance factor Random Forest analysis (Im RF), not determined (n.d), Serum Amyloid P (SAP), Prostatic Acid Phosphatase (PAP), Macrophage Inflammatory Protein-1 beta (MIP1b), Complement 3 (C3), Fatty Acid Binding Protein (FABP), Acylation Stimulating Protein (ASP), Vascular endothelial growth factor (VEGF), Creatine Kinase-MB (CK-MB), Tumor Necrosis Factor alpha (TNFα), Myeloperoxidase (MPO), Pancreatic Polypeptide (PP), Apolipoprotein CIII (Apo CIII), Epithelial neutrophil-activating peptide 78 (ENA-78), C Reactive Protein (CRP).

In population II, 9 out of the 20 selected proteins in population I were associated with plasma insulin concentrations (table 3, figure 3B and figure S1). As for BMI associations with insulin in population II were more robust compared to associations with insulin in population I.

### Pathway analysis

**Figure 4:**
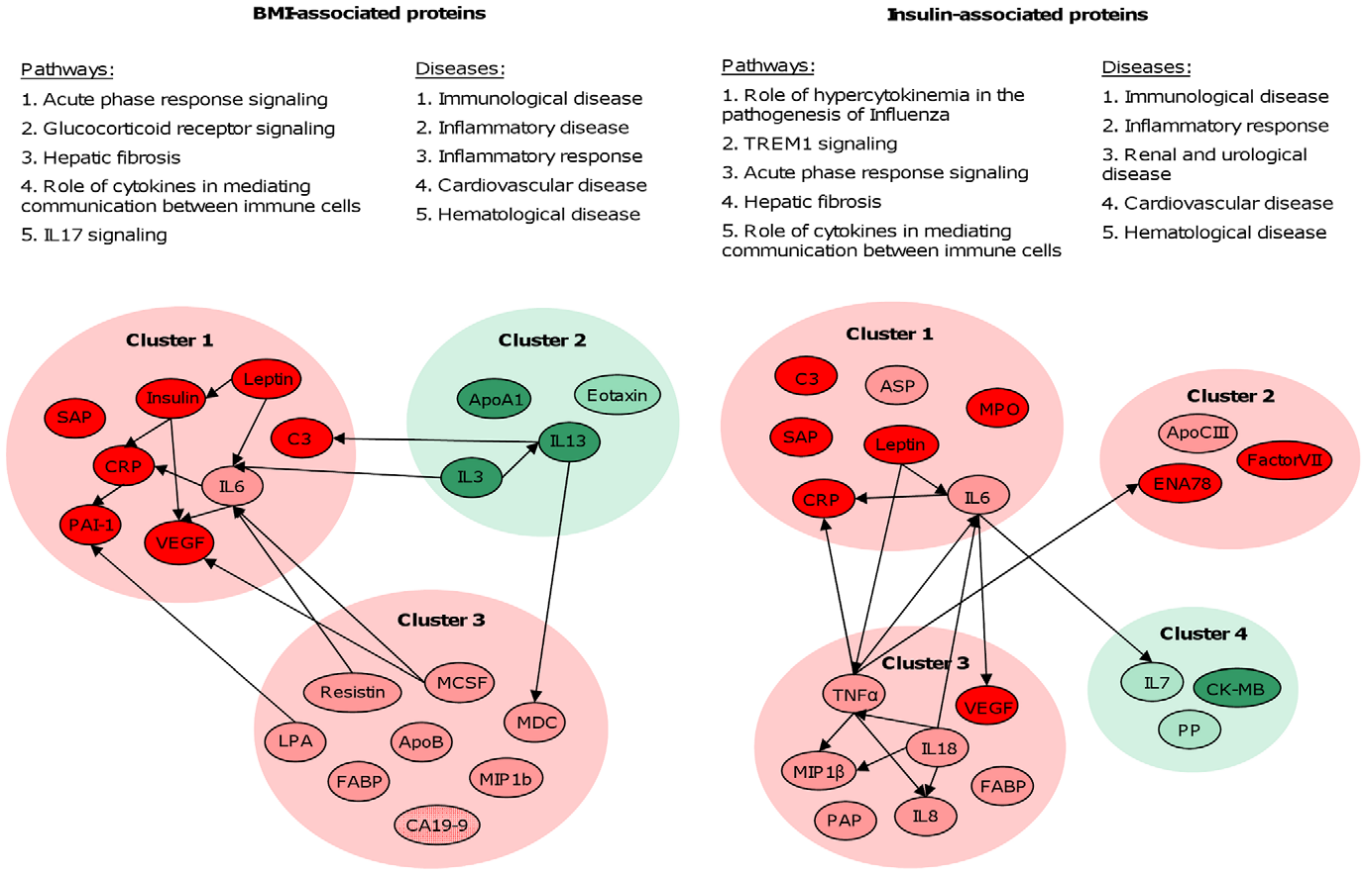
BMI- and insulin-associated proteins, their interactions and pathways and diseases in which they are involved. All defined clusters of BMI and insulin-associated proteins are displayed, in red the positively associated clusters and in green the negatively associated clusters. In dark red or green BMI and insulin-associated proteins in both populations are shown, in light red and light green only the BMI and insulin-associated proteins in population I. Connections between proteins and the top 5 canonical pathways and diseases relevant to the data are shown. Abbreviations: Triggering Receptor Expressed on Myeloid cells 1 (TREM1).

An overview of all clusters of proteins and interactions between the single proteins selected by RF is displayed in figure 4. This figure also shows the top 5 most significant pathways and diseases for the BMI- and insulin-associated proteins. Acute phase response signaling was the most significant pathway for BMI-associated proteins and was also significant for insulin-associated proteins.

## Discussion

Elevated plasma levels of several pro-inflammatory proteins are related to the development of obesity-linked diseases, in particular to T2DM and CVD [4], [20], [21], [22]. In the current study we observed associations of clusters of pro- and anti-inflammatory proteins with BMI and insulin in a presumably healthy population. These BMI- and insulin-associated protein clusters may serve as biomarkers for a pre-disease state of people at risk to develop CVD and T2DM. The protein clusters could possibly be used to improve individual disease risk prediction and help in the design of personalized strategies to prevent disease as early as possible.

The protein cluster showing the most robust positive association with BMI contained a number of pro-inflammatory proteins of which several are involved in the acute phase response, such as CRP, IL-6, C3 and SAP. These and other proteins (i.e. ASP, MPO, PAI-1 and VEGF) included in this cluster and in cluster 1 that was associated with insulin were previously found to be increased in subjects with insulin resistance, CVD, or both [3], [21], [23], [24], [25], [26], [27], [28], [29], [30], [31], [32]. From prospective studies evidence has accumulated that increased levels of C3 and CRP can predict T2DM and coronary events and could be candidate biomarkers for a pathological state preceding the ultimate disease [3], [8], [26], [27]. We hypothesize that the proteins clustering together with CRP and C3 could be similar type of biomarkers. Moreover, Macrophage Colony-Stimulating Factor (MCSF) which was highly positively associated with BMI was discovered to be a prognostic marker of cardiovascular events in patients with chronic coronary artery disease [33]. We speculate that MCSF may also be an early biomarker for cardiac disease in healthy subjects.

The positive correlations of BMI with leptin and other adipose-tissue derived proteins, as seen in our study, supports the view of adipose tissue as an important source of immune-related proteins [34]. However, besides adipose tissue-derived proteins also proteins produced by the liver, endothelial cells, immune cells and lipoproteins were associated with BMI and insulin in our healthy subjects. This indicates that low-grade inflammation in the early disease state is not only an adipose tissue-specific effect but that also organ cells increase their secretion of inflammatory proteins with increasing BMI or plasma insulin levels.

A small cluster of proteins (ApoA1, CA19-9, Eotaxin, IL-3 and IL-13) was negatively associated with BMI. Plasma levels of tumor marker CA19-9 and ApoA1, the major protein component of plasma HDL, were reported to be lower in obesity [35], [36]. Less is known about the negative association of BMI with Th2 cytokines IL-3 and IL-13 and Eotaxin, which are correlated with each other and closely clustered [37], [38], [39]. Concentrations of IL-3 and IL-13 were on average very low in the populations so we should be careful with the interpretation of this data. Nevertheless we observed associations of IL-3 and IL-13 with BMI in both study populations, therefore these proteins might be considered as potential new early biomarkers for obesity-related diseases.

Associations between strongly associated protein clusters 1 and 2 with BMI and insulin in the primary study population were confirmed in the second population. Weaker associations were not all confirmed which could be attributable to the lower number of subjects in population II and the fact that less proteins were measured reducing the chance of finding protein interactions with RF. However, the strong associations found in population I were even more pronounced in population II. The latter population was significantly older but concentrations of individual plasma pro-inflammatory proteins were not higher. Maybe an elevated BMI or insulin concentration in elderly subjects is directly linked to an increase in pro-inflammatory protein secretion while this is not always the case in younger subjects who may display a more flexible metabolic phenotype in handling changes in BMI or insulin concentration.

Levels of individual proteins were not extremely elevated in our study subjects, but based on protein profiles subjects may be differentially classified with lower or higher risk to develop a more pathological phenotype. Despite the fact that the risk profile was BMI-related in the whole study population, there were a few subjects with low BMI also having this risk profile and a few subjects with high BMI not having the profile. Besides BMI, waist-to-height ratio can be used as a determinant for obesity-related health risk. When the same analyses were performed for the association of proteins with waist-to-height ratio similar results as for BMI were found. Our findings support the recent ideas of using a ‘multimarker’ approach, i.e. measuring multiple plasma proteins in instead of single biomarkers or only BMI to increase the prognostic value for individual disease risk [8], [40], [41].

Still, the measurement of multiple proteins makes data analysis complex and calls for more advanced methods of data analysis. Using RF we were able to identify associations of proteins with BMI or insulin that were not observed when associations of each protein were analyzed separately, but combined with other proteins may be of relevance. For RF we chose a liberal threshold for selection of proteins associated with BMI or insulin, which increases the risk of selecting proteins that are not related to the outcome of interest. However, using this threshold, we observed a high overlap between the results from RF and univariate analysis. Furthermore, we applied this analysis to two independent populations to replicate and verify the associations of protein clusters with BMI and insulin. In our view, this approach has increased the reliability of our results.

The results of our study are based on a single measurement of plasma proteins that provides a “snapshot” of the actual health status. Whether it can be used in predicting long-term health and inflammatory status requires more long-term studies. Furthermore, our primary study population consisted of a relatively low number of subjects. However, we have observed associations of proteins with BMI or insulin that are physiologically relevant and were found in epidemiological and clinical studies [3], [5], [6], [8], [21], [23], [24], [31], [32]. Moreover, we were able to confirm the most robust associations in a second, even smaller population consisting of subjects from a different age group.

With our study it is not possible to draw conclusions about the causal relationship between elevated concentrations of the selected proteins and occurrence of disease. Further prospective studies with clinical end points are needed to determine whether the protein clusters found in this study could be used as reliable biomarkers for early identification of persons at risk for T2DM and CVD.

Our study aimed at identifying new leads for clusters of early biomarkers of disease by using plasma protein profiling in healthy subjects. In healthy subjects we identified clusters of proteins associated with BMI and insulin that included previously identified biomarkers for obesity-related disease risk and potential new biomarkers for which an association with disease is not well-established. We showed that plasma protein profiling allows a more subtle phenotypic characterization and differentiation of people with otherwise similar phenotypical features such as BMI or insulin levels. This could be of great value for dietary and pharmacological intervention studies where subgroups of volunteers with matching phenotypes could be included in order to improve the power of such interventions. Improved individual risk assessment and classification of subjects may ultimately lead to a more tailored and adequate intervention either by pharmacology or changes in lifestyle.

## Supporting Information

Table S1 Proteins included in the analysis for both populations. ‘+’ indicates that the protein is measured and detected in more than half of the samples and included in the analysis. ‘-’ indicates that the protein is not measured or not detected in more than half of the samples and therefore not included in the analysis.
(0.14 MB DOC)

Table S2 Protein concentrations in each population Concentrations are displayed as mean ± standard deviation. Abbreviations: not determined (n.d.)
(0.14 MB DOC)

Figure S1 Overview of selection of proteins for analysis in both populations
(0.12 MB TIF)
