# Supplementary material for: Plasma Protein Profiling Reveals Protein Clusters Related to BMI and Insulin Levels in Middle-Aged Overweight Subjects
Source: PLoS One. 2010 Dec 23;5(12):e14422. doi: 10.1371/journal.pone.0014422 (PMC3009718; doi:10.1371/journal.pone.0014422)
Supplement: Table S1 — Proteins included in the analysis for both populations. ‘+’ indicates that the protein is measured and detected in more than half of the samples and included in the analysis. ‘-’ indicates that the protein is not measured or not detected in more than half of the samples and therefore not included in the analysis. (0.14 MB DOC) [file pone.0014422.s001.doc]

| **Protein** | **Population I** | **Population II** |
| --- | --- | --- |
| 1. Alpha-1 Antitrypsin | **+** | **+** |
| 2. Angiotensin Converting Enzyme (ACE) | **+** | **+** |
| 3. Adrenocorticotropic Hormone (ACTH) | **+** | **-** |
| 4. Adiponectin | **+** | **+** |
| 5. Alpha-2-Macroglobulin (A2M) | **+** | **+** |
| 6. Alpha Fetoprotein | **+** | **+** |
| 7. Amphiregulin | - | - |
| 8. Angiotensinogen | **+** | **+** |
| 9. Apolipoprotein A1 (Apo A1) | **+** | **+** |
| 10. Apolipoprotein CIII (Apo CIII) | **+** | **+** |
| 11. Apolipoprotein H (Apo H) | **+** | **+** |
| 12. Acylation Stimulating Protein (ASP, C3 des Arg) | + | + |
| 13. Beta-2 Microglobulin (B2M) | + | + |
| 14. Betacellulin | + | - |
| 15. Brain-Derived Neurotropic Factor (BDNF) | + | + |
| 16. Complement 3 (C3) | + | + |
| 17. Cancer Antigen 125 (CA-125) | + | - |
| 18. Cancer Antigen 19-9 (CA-19-9) | + | + |
| 19. Calcitonin | + | - |
| 20. CD40 | **+** | **+** |
| 21. CD40 Ligand (CD40L) | + | + |
| 22. Carcinoembryonic Antigen (CEA) | + | + |
| 23. Creatine Kinase-MB (CK-MB) | + | + |
| 24. Cortisol | + | + |
| 25. C Reactive Protein (CRP) | + | + |
| 26. Connective Tissue Growth Factor (CTGF) | + | - |
| 27. Epidermal Growth Factor (EGF) | + | + |
| 28. Epidermal Growth Factor Receptor (EGFR) | + | - |
| 29. Epithelial cell-derived neutrophil-activating peptide 78 (ENA-78) | + | + |
| 30. Endothelin-1 (ET-1) | + | + |
| 31. Extracellular rage binding protein (EN-RAGE) | + | + |
| 32. Eotaxin (CCL 11) | + | + |
| 33. Epiregulin | + | - |
| 34. Erythropoietin | + | + |
| 35. Fatty Acid Binding Protein (FABP) | + | + |
| 36. Factor VII | + | + |
| 37. Ferritin | + | + |
| 38. Fibroblast Growth Factor Basic (bFGF) | + | + |
| 39. Fibrinogen | + | + |
| 40. Follicle Stimulating Hormone (FSH) | + | + |
| 41. Galanin | - | - |
| 42. Granulocyte colony-stimulating factor (GCSF) | - | - |
| 43. Growth Hormone (GH) | + | + |
| 44. Glucagon-like peptide-1 active (GLP1 active) | + | - |
| 45. Glucagon-like peptide**-**1 Total (GLP1 total) | + | + |
| 46. Glucagon | + | + |
| 47. Granulocyte-macrophage colony-stimulating factor (GM-CSF) | - | - |
| 48. Glutathione S-Transferase (GST) | + | + |
| 49. Haptoglobin | + | + |
| 50. Heparin-Binding Epidermal Growth Factor (HB-EGF) | + | - |
| 51. Inter-Cellular Adhesion Molecule 1 (ICAM 1) | + | + |
| 52. Interferon gamma (IFN-γ) | - | - |
| 53. Immunoglobulin A (IgA) | + | + |
| 54. Immunoglobulin E (IgE) | + | + |
| 55. Insulin-like growth factor (IGF) | - | - |
| 56. Immunoglobulin M (IgM) | + | + |
| 57. Interleukin-10 (IL10) | + | + |
| 58. Interleukin-11 (IL11) | + | - |
| 59. Interleukin-12p40 (IL12p40) | - | - |
| 60. Interleukin-12p70 (IL12p70) | + | + |
| 61. Interleukin-13 (IL13) | + | + |
| 62. Interleukin-15 (IL15) | + | + |
| 63. Interleukin-16 (IL16) | + | + |
| 64. Interleukin-17 (IL17) | + | - |
| 65. Interleukin-17E (IL17E) | - | - |
| 66. Interleukin-18 (IL18) | + | + |
| 67. Interleukin-1alpha (IL1a) | - | - |
| 68. Interleukin-1 beta (IL1b) | + | - |
| 69. Interleukin-1 receptor antagonist (IL1RA) | + | + |
| 70. Interleukin-2 (IL2) | - | - |
| 71. Interleukin-23 (IL23 | + | - |
| 72. Interleukin-3 (IL3) | + | + |
| 73. Interleukin-4 (IL4) | + | + |
| 74. Interleukin-5 (IL5) | + | + |
| 75. Interleukin-6 (IL6) | - | - |
| 76. Interleukin-7 (IL7) | + | + |
| 77. Interleukin-8 (IL8) | + | + |
| 78. Insulin | + | + |
| 79. Leptin | + | + |
| 80. Luteinizing Hormone (LH) | + | + |
| 81. Lipoprotein a (LPA) | + | + |
| 82. Lymphotactin | - | - |
| 83. Monocyte Chemotactic Protein-1 (MCP1) | + | + |
| 84. Macrophage colony-stimulating factor (MCSF) | + | - |
| 85. Macrophage Derived Chemokine (MDC) | + | + |
| 86. Macrophage Inflammatory Protein 1 alpha (MIP1a/ CCL3) | + | + |
| 87. Macrophage Inflammatory Protein 1 beta (MIP1b/ CCL4) | + | + |
| 88. Matrix Metalloproteinase-2 (MMP2) | + | + |
| 89. Matrix Metalloproteinase-3 (MMP3) | + | - |
| 90. Matrix Metalloproteinase-9 (MMP9) | + | + |
| 91. Myeloperoxidase (MPO) | + | + |
| 92. Myoglobin | + | + |
| 93. Oncostatin M (OSM) | - | - |
| 94. Plasminogen activator inhibitor-1 (PAI-1) | + | + |
| 95. Pancreatic polypeptide (PP) | + | + |
| 96. Prostatic Acid Phosphatase (PAP) | + | + |
| 97. Pregnancy-associated plasma protein A (PAPPA) | + | + |
| 98. Platelet-derived growth factor (PDGF) | + | - |
| 99. Progesterone | + | + |
| 100. Prolactin | + | + |
| 101. Prostate Specific Antigen (PSA) | + | + |
| 102. Peptide YY (PYY) | + | + |
| 103. Regulated on Activation Normal T Cell Expressed and Secreted (RANTES/ CCL5) | + | + |
| 104. Resistin | + | + |
| 105. Serum Amyloid P (SAP) | + | + |
| 106. Stem Cell Factor (SCF) | + | + |
| 107. Secretin | - | - |
| 108. Serum glutamic oxaloacetic transaminase (SGOT) | + | + |
| 109. Sex Hormone Binding Globulin (SHBG) | + | + |
| 110. Thyroxine Binding Globulin (TBG) | + | + |
| 111. Tenascin C | + | - |
| 112. Testosterone | + | + |
| 113. Tissue Factor (TF) | + | + |
| 114. Transforming growth factor-alpha (TGFa) | - | - |
| 115. Tissue inhibitor of metalloproteinase 1 (TIMP1) | + | + |
| 116. Tumour necrosis factor alpha receptor type II (TNFaRII) | + | + |
| 117. Tumour necrosis factor alpha (TNFa) | + | + |
| 118. Tumour necrosis factor beta (TNFb) | - | - |
| 119. Thrombopoietin (TPO) | + | + |
| 120. Thyroid Stimulating Hormone (TSH) | + | + |
| 121. Thrombospondin-1 (THBS1) | + | - |
| 122. Vascular cell adhesion molecule-1 (VCAM1) | + | + |
| 123. Vascular endothelial growth factor (VEGF) | + | + |
| 124. von Willebrand Factor (vWF) | + | + |
