# Supplementary material for: Plasma Protein Profiling Reveals Protein Clusters Related to BMI and Insulin Levels in Middle-Aged Overweight Subjects
Source: PLoS One. 2010 Dec 23;5(12):e14422. doi: 10.1371/journal.pone.0014422 (PMC3009718; doi:10.1371/journal.pone.0014422)
Supplement: Table S2 — Protein concentrations in each population Concentrations are displayed as mean ± standard deviation. Abbreviations: not determined (n.d.) (0.14 MB DOC) [file pone.0014422.s002.doc]

**Table S2** Protein concentrations in each population

| **Protein** | **Population I** | **Population II** |
| --- | --- | --- |
|
| Alpha-1 Antitrypsin (mg/mL) | 1.99 ± 0.31 | 1.76 ± 0.33 |
| Angiotensin Converting Enzyme (ACE) (ng/mL) | 118 ± 35 | 154 ± 52 |
| Adrenocorticotropic Hormone (ACTH) (ng/mL) | 0.18 ± 0.15 | n.d. |
| Adiponectin (μg/mL) | 4.39 ± 2.67 | 3.45 ± 1.69 |
| Alpha-2-Macroglobulin (A2M) (mg/mL) | 0.84 ± 0.19 | 0.9 ± 0.11 |
| Alpha Fetoprotein (ng/mL) | 3.87 ± 1.64 | 2.80 ± 1.65 |
| Angiotensinogen (ng/mL) | 8.73 ± 15.79 | 5.35 ± 5.13 |
| Apolipoprotein A1 (Apo A1) (mg/mL) | 0.31 ± 0.10 | 0.27 ± 0.09 |
| Apolipoprotein CIII (Apo CIII) (μg/mL) | 93.8 ± 24.7 | 85.7 ± 20.3 |
| Apolipoprotein H (Apo H) (μg/mL) | 214 ± 45 | 198 ± 38 |
| Acylation Stimulating Protein (ASP, C3 des Arg) (ng/mL) | 1835 ± 398 | 2625 ± 198 |
| Beta-2 Microglobulin (B2M) (μg/mL) | 1.65 ± 0.34 | 1.79 ± 0.55 |
| Betacellulin (pg/mL) | 122.9 ± 88.8 | n.d. |
| Brain-Derived Neurotropic Factor (BDNF) (ng/mL) | 1.99 ± 2.96 | 0.03 ± 0.04 |
| Complement 3 (C3) (mg/mL) | 0.95 ± 0.15 | 0.85 ± 0.12 |
| Cancer Antigen 125 (CA-125) (U/mL) | 9.37 ± 7.21 | n.d. |
| Cancer Antigen 19-9 (CA-19-9) (U /mL) | 6.96 ± 6.03 | 7.18 ± 5.80 |
| Calcitonin (pg/mL) | 8.38 ± 3.52 | n.d. |
| CD40 (ng/mL) | 0.65 ± 0.14 | 0.83 ± 0.37 |
| CD40 Ligand (CD40L) (ng/mL) | 0.24 ± 0.22 | 0.05 ± 0.03 |
| Carcinoembryonic Antigen (CEA) (ng/mL) | 1.50 ± 1.13 | 1.61 ± 0.86 |
| Creatine Kinase-MB (CK-MB) (ng/mL) | 0.38 ± 0.22 | 0.61 ± 0.34 |
| Cortisol (ng/mL) | 119 ± 44 | 124 ± 58 |
| C Reactive Protein (CRP) (μg/mL) | 3.00 ± 4.57 | 4.45 ± 6.61 |
| Connective Tissue Growth Factor (CTGF) (ng/mL) | 2.24 ± 1.01 | n.d. |
| Epidermal Growth Factor (EGF) (pg/mL) | 207 ± 116 | 22 ± 6 |
| Epidermal Growth Factor Receptor (EGFR) (ng/mL) | 142 ± 33 | n.d. |
| Epithelial cell-derived neutrophil-activating peptide 78 (ENA-78) (ng/mL) | 1.10 ± 1.10 | 0.06 ± 0.06 |
| Endothelin-1 (ET-1) (pg/mL) | 23.5 ± 11.7 | 10.3 ± 9.7 |
| Extracellular rage binding protein (EN-RAGE) (ng/mL) | 7.4 ± 6.3 | 2.3 ± 1.3 |
| Eotaxin (CCL 11) (pg/mL) | 133 ± 81 | 80 ± 54 |
| Epiregulin (pg/mL) | 23.7 ± 27.8 | n.d. |
| Erythropoietin (pg/mL) | 84 ± 83 | 64 ± 90 |
| Fatty Acid Binding Protein (FABP) (ng/mL) | 1.64 ± 1.29 | 2.37 ± 1.58 |
| Factor VII (ng/mL) | 534 ± 96 | 651 ± 183 |
| Ferritin (ng/mL) | 147 ± 117 | 140 ± 94 |
| Fibroblast Growth Factor Basic (bFGF) (pg/mL) | 155 ± 143 | 92 ± 76 |
| Fibrinogen (mg/mL) | 4.5 ± 0.9 | 3.1 ± 0.7 |
| Follicle Stimulating Hormone (FSH) (ng/mL) | 15.2 ± 8.7 | 14.4 ± 9.6 |
| Growth Hormone (GH) (ng/mL) | 1.24 ± 1.87 | 3.01 ± 4.36 |
| Glucagon-like peptide-1 active (GLP1 active) (pg/mL) | 17.96 ± 4.62 | n.d. |
| Glucagon-like peptide-1 Total (GLP1 total) (pg/mL) | 74.74 ± 34.61 | n.d. |
| Glucagon (pg/mL) | 651 ± 226 | 623 ± 260 |
| Glutathione S-Transferase (GST) (ng/mL) | 1.01 ± 0.33 | 0.75 ± 0.09 |
| Haptoglobin (mg/mL) | 1.41 ± 0.78 | 1.48 ± 0.67 |
| Heparin-Binding Epidermal Growth Factor (HB-EGF) (pg/mL) | 92.8 ± 44.1 | n.d. |
| Inter-Cellular Adhesion Molecule 1 (ICAM 1) (ng/mL) | 140 ± 45 | 133 ± 34 |
| Immunoglobulin A (IgA) (mg/mL) | 1.43 ± 0.58 | 1.05 ± 0.35 |
| Immunoglobulin E (IgE) (ng/mL) | 106.4 ± 153.0 | 151.8 ± 268.7 |
| Immunoglobulin M (IgM) (mg/mL) | 1.08 ± 0.58 | 1.12 ± 0.83 |
| Interleukin-10 (IL10) (pg/mL) | 9.81 ± 3.59 | 11.24 ± 1.37 |
| Interleukin-11 (IL11) (pg/mL) | 61.0 ± 93.6 | n.d. |
| Interleukin-12p70 (IL12p70) (pg/mL) | 70.4 ± 32.0 | 43.8 ± 3.2 |
| Interleukin-13 (IL13) (pg/mL) | 56.9 ± 14.0 | 67.4 ± 14.0 |
| Interleukin-15 (IL15) (ng/mL) | 0.32 ± 0.10 | 0.39 ± 0.08 |
| Interleukin-16 (IL16) (pg/mL) | 441 ± 189 | 318 ± 75 |
| Interleukin-17 (IL17) (pg/mL) | 24.2 ± 4.8 | n.d. |
| Interleukin-18 (IL18) (pg/mL) | 239 ± 78 | 197 ± 120 |
| Interleukin-1 beta (IL1b) (ng/mL) | 1.96 ± 1.82 | n.d. |
| Interleukin-1 receptor antagonist (IL1RA) (pg/mL) | 100.8 ± 60.8 | 88.9 ± 69.1 |
| Interleukin-23 (IL23) (ng/mL) | 0.85 ± 0.82 | n.d. |
| Interleukin-3 (IL3) (ng/mL) | 0.07 ± 0.05 | 0.04 ± 0.04 |
| Interleukin-4 (IL4) (pg/mL) | 33.5 ± 9.7 | 61.2 ± 20.3 |
| Interleukin-5 (IL5) (pg/mL) | 10.87 ± 14.44 | 5.20 ± 2.62 |
| Interleukin-6 (IL6) (pg/mL) | 1.30 ± 1.67 | n.d. |
| Interleukin-7 (IL7) (pg/mL) | 63.0 ± 18.1 | 60.4 ± 12.6 |
| Interleukin-8 (IL8) (pg/mL) | 17.8 ± 7.3 | 12.7 ± 4.5 |
| Leptin (ng/mL) | 14.17 ± 15.95 | 15.33 ± 16.70 |
| Luteinizing Hormone (LH) (ng/mL) | 1.63 ± 0.93 | 0.94 ± 0.79 |
| Lipoprotein a (LPA) (μg/mL) | 163.9 ± 180.8 | 131.4 ± 121.1 |
| Monocyte Chemotactic Protein-1 (MCP1) (pg/mL) | 100.3 ± 68.5 | 126.4 ± 43.6 |
| Macrophage colony-stimulating factor (MCSF) (ng/mL) | 7.27 ± 4.60 | n.d. |
| Macrophage Derived Chemokine (MDC) (pg/mL) | 379 ± 86 | 301 ± 67 |
| Macrophage Inflammatory Protein 1 alpha (MIP1a/ CCL3) (pg/mL) | 57.8 ± 9.7 | 106.1 ± 19.6 |
| Macrophage Inflammatory Protein 1 beta (MIP1b/ CCL4) (pg/mL) | 168 ± 106 | 136 ± 48 |
| Matrix Metalloproteinase-2 (MMP2) (ng/mL) | 2093 ± 400 | 2373 ± 461 |
| Matrix Metalloproteinase-3 (MMP3) (ng/mL) | 0.07 ± 0.09 | n.d. |
| Matrix Metalloproteinase-9 (MMP9) (ng/mL) | 110.5 ± 119.5 | 49.4 ± 55.7 |
| Myeloperoxidase (MPO) (ng/mL) | 142.1 ± 54.0 | 96.7 ± 45.4 |
| Myoglobin (ng/mL) | 15.01 ± 19.58 | 19.71 ± 8.16 |
| Plasminogen activator inhibitor-1 (PAI-1) (pg/mL) | 45.7 ± 27.4 | 30.0 ± 19.7 |
| Pancreatic polypeptide (PP) (pg/mL) | 192 ± 120 | 193 ± 67 |
| Prostatic Acid Phosphatase (PAP) (ng/mL) | 0.29 ± 0.23 | 0.19 ± 0.08 |
| Pregnancy-associated plasma protein A (PAPPA) (mIU/mL) | 0.04 ± 0.01 | 0.05 ± 0.04 |
| Platelet-derived growth factor (PDGF) (pg/mL) | 2088 ± 1452 | n.d. |
| Progesterone (ng/mL) | 7.72 ± 23.40 | 3.49 ± 1.40 |
| Prolactin (ng/mL) | 15.2 ± 6.1 | 12.9 ± 7.7 |
| Prostate Specific Antigen (PSA) (ng/mL) | 0.46 ± 0.84 | 0.41 ± 0.65 |
| Peptide YY (PYY) (pg/mL) | 61.3 ± 35.4 | 52.1 ± 39.8 |
| Regulated on Activation Normal T Cell Expressed and Secreted (RANTES/ CCL5) (ng/mL) | 8.12 ± 9.05 | 0.31 ± 0.16 |
| Resistin (ng/mL) | 3.28 ± 0.94 | 2.28 ± 0.60 |
| Serum Amyloid P (SAP) (μg/mL) | 18.6 ± 5.3 | 17.3 ± 3.5 |
| Stem Cell Factor (SCF) (pg/mL) | 217 ± 74 | 209 ± 52 |
| Serum glutamic oxaloacetic transaminase (SGOT) (μg/mL) | 15.6 ± 2.7 | 10.2 ± 2.1 |
| Sex Hormone Binding Globulin (SHBG) (nmol/L) | 44.7 ± 21.1 | 33.7 ± 11.1 |
| Thyroxine Binding Globulin (TBG) (μg/mL) | 54.3 ± 13.5 | 43.5 ± 8.7 |
| Tenascin C (ng/mL) | 1323 ± 767 | n.d. |
| Testosterone (ng/mL) | 1.50 ± 1.16 | 1.70 ± 1.17 |
| Tissue Factor (TF) (ng/mL) | 0.65 ± 0.31 | 0.49 ± 0.13 |
| Tissue inhibitor of metalloproteinase 1 (TIMP1) (ng/mL) | 82.6 ± 17.3 | 80.7 ± 11.4 |
| Tumour necrosis factor alpha receptor type II (TNFaRII) (ng/mL) | 3.41 ± 0.91 | 4.14 ± 1.01 |
| Tumour necrosis factor alpha (TNFa) (pg/mL) | 7.60 ± 2.18 | 5.35 ± 2.04 |
| Thrombopoietin (TPO) (ng/mL) | 1.38 ± 1.09 | 0.86 ± 0.57 |
| Thyroid Stimulating Hormone (TSH) (μIU/mL) | 4.14 ± 7.67 | 4.22 ± 9.43 |
| Thrombospondin-1 (THBS1) (ng/mL) | 8111 ± 8583 | n.d. |
| Vascular cell adhesion molecule-1 (VCAM1) (ng/mL) | 555 ± 105 | 571 ± 78 |
| Vascular endothelial growth factor (VEGF) (pg/mL) | 604 ± 146 | 510 ± 143 |
| von Willebrand Factor (vWF) (μg/mL) | 29.9 ± 11.5 | 11.6 ± 10.4 |
