# Supplementary figures and images for: Plasma Protein Profiling Reveals Protein Clusters Related to BMI and Insulin Levels in Middle-Aged Overweight Subjects
Source: PLoS One. 2010 Dec 23;5(12):e14422. doi: 10.1371/journal.pone.0014422 (PMC3009718; doi:10.1371/journal.pone.0014422)

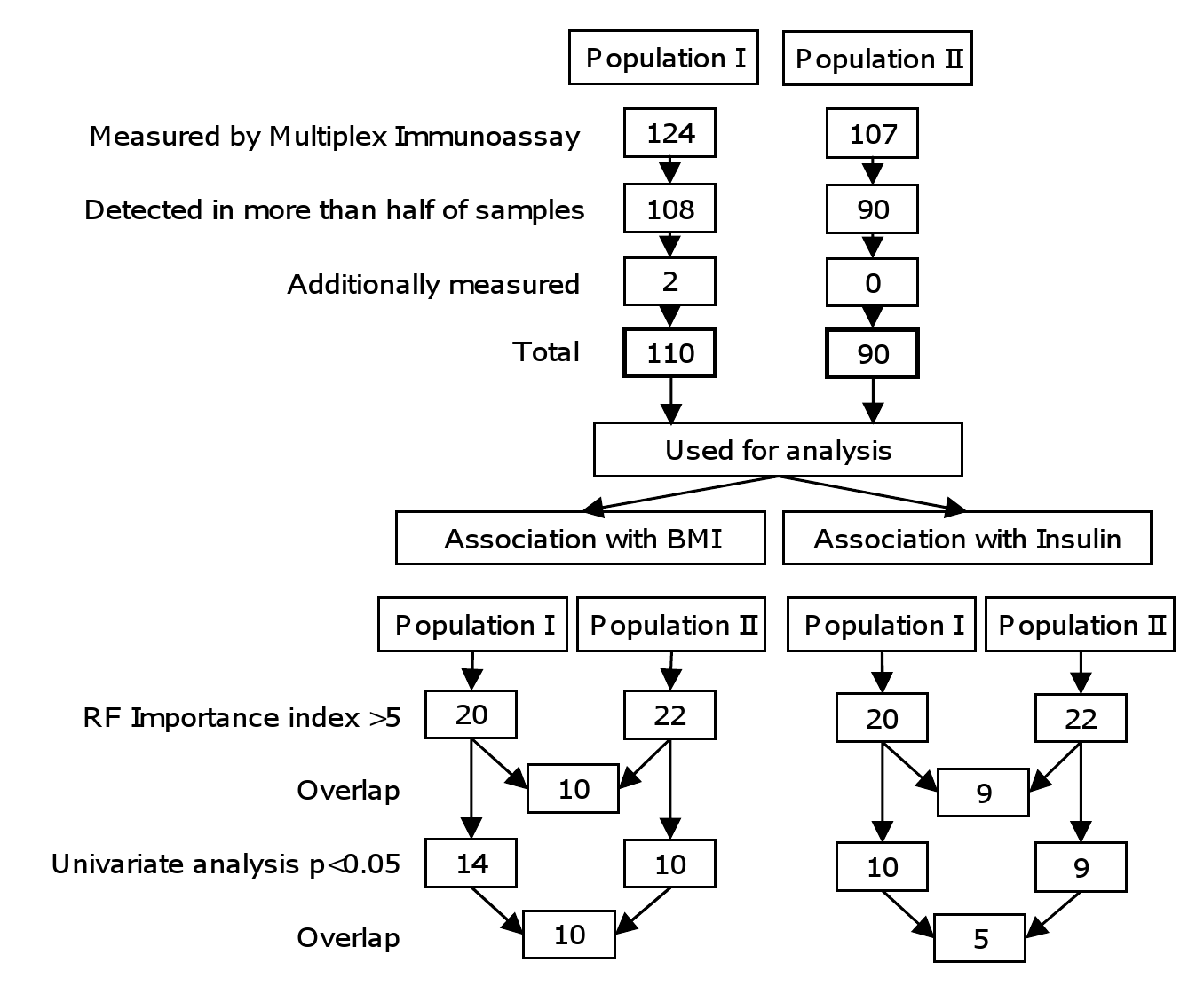

Supplement: Figure S1 — Overview of selection of proteins for analysis in both populations (0.12 MB TIF) [file pone.0014422.s003.tif]
